# 3D imaging and morphometric descriptors of vascular networks on optically cleared organs

**DOI:** 10.1016/j.isci.2023.108007

**Published:** 2023-09-22

**Authors:** Nabil Nicolas, Virginie Dinet, Etienne Roux

**Affiliations:** 1 University Bordeaux, INSERM, Biologie des maladies cardiovasculaires, U1034, F-33600 Pessac, France

**Keywords:** Imaging anatomy, Optical imaging

## Abstract

The vascular system is a multi-scale network whose functionality depends on its structure, and for which structural alterations can be linked to pathological shifts. Though biologists use multiple 3D imaging techniques to visualize vascular networks, the 3D image processing methodologies remain sources of biases, and the extraction of quantitative morphometric descriptors remains flawed. The article, first, reviews the current 3D image processing methodologies, and morphometric descriptors of vascular network images mainly obtained by light-sheet microscopy on optically cleared organs, found in the literature. Second, it proposes operator-independent segmentation and skeletonization methodologies using the freeware ImageJ. Third, it gives more extractable network-level (density, connectivity, fractal dimension) and segment-level (length, diameter, tortuosity) 3D morphometric descriptors and how to statistically analyze them. Thus, it can serve as a guideline for biologists using 3D imaging techniques of vascular networks, allowing the production of more comparable studies in the future.

## Graphical abstract

**Figure:**
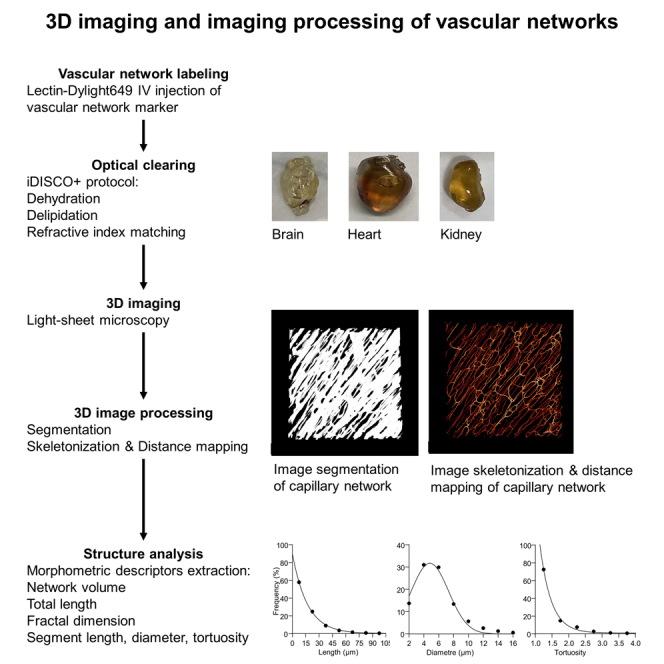

## Introduction

The vascular system is constituted of the blood vessels (arteries, capillaries and veins) organized in complex connected networks through which circulates the blood, ensuring perfusion and diffusive exchanges in the body. The functional efficiency of the vascular system critically depends on its architecture, and many diseases are related to vascular dysfunction. Hence, the morphometric description of this architecture is important to characterize the normal vascular network 3D structure and identify their pathophysiological alterations. Morphometric descriptors are parameters that provide a quantitative information regarding the morphology of the vascular network, hence allowing the statistical comparison of the vascular architecture between samples. A variety of imaging techniques of the vessels have been developed through time, with 3D imaging techniques such as microtomodensitometry and, more recently, light sheet microscopy (LSM) on optically cleared organs. Despite these technical innovations allowing the visualization of the 3D structure of these networks and the availability of image processing computational tools, extraction of 3D morphometric descriptors remains, nowadays, an important limit in 3D studies of vascular networks. The aim of this article is to provide the basic concepts for the quantitative characterization of the vascular network structure and operational practical methodologies for 3D image processing and analysis up to the extraction of 3D morphometric descriptors (MDs).

The article focuses on the analysis of the capillary network imaged by LSM on optically cleared organs, but the application of the concepts and methods to other structures (e.g., arterial network) and imaging techniques (e.g., X-ray microscanner) is discussed. The main methods and tools used at the different steps of image processing and analysis are presented with their basic principles, advantages, and limitations, with special attention to ImageJ freeware functions and plugins and our own methodology, hence accessible to any biologist without specialized mathematical or computational expertise. The definition, meaning, and calculation of the main MDs are given and illustrated with examples taken from the literature and our own results.

## Vascular networks

**Figure 1:**
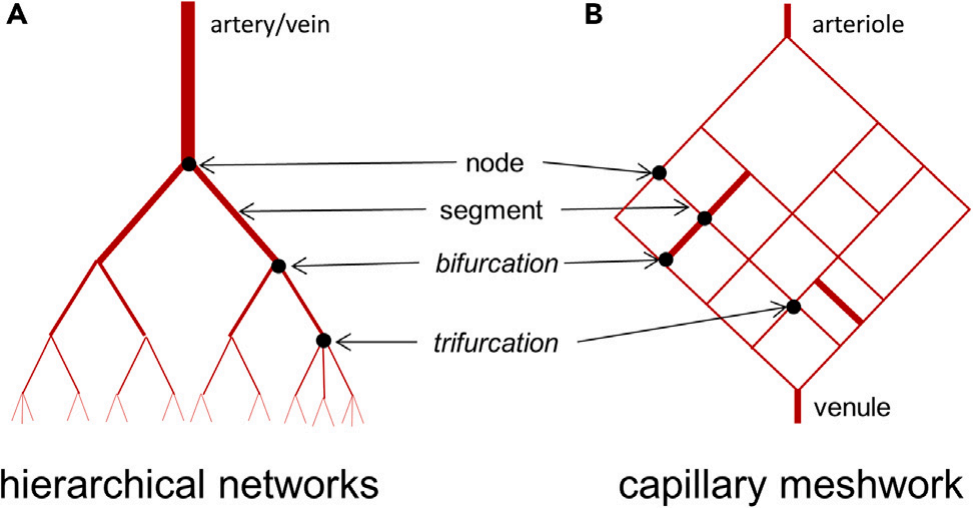
Schematization of arterial and capillary network (A) Arborescent arterial network. (B) Meshworked capillary network.

The vascular system is a multi-scale network that can be schematically represented by 3 connected networks characterized by 2 different network patterns. The arteries and veins are branched, hierarchically organized networks with successive furcations from the aorta to the precapillary arterioles and the post-capillary venules to the vena cava (Figure 1A), respectively, whereas the capillaries, joining arterioles to venules, constitute a non-hierarchical meshwork (Figure 1B). Some MDs are hence relevant just for one type of networks, e.g., the hierarchical order of the segments depending on their position in a tree-like network. However, both network types share common pattern properties, notably the fact that they can be considered as fractal, i.e., self-similar structures built by the reproduction of the same basic pattern at different scales, and can hence be characterized by the same MDs. MDs, their extraction from the images, and statistical analysis are detailed in a dedicated section.

## Three-dimensional imaging

### Optical imaging

Quantitative characterization of a vascular network implies a good visualization of it. Many techniques are available for the 3D visualization of vascular networks. One of the most efficient techniques is the combination of optical clearing with 3D light microscopy. Optical clearing of an organ consists in homogenizing the organ refractive index, i.e., reducing the light refractive capacity of the different tissues composing the organ, usually by removing the lipids, in order to let it become transparent when immersed in a solution with the same refractive index.^1^ As described in 2019 by Costa and coauthors, there an increasing number of optical clearing protocols (such as iDisco, Clarity, Cubic, BABB, and so forth) that can be classified in four types of methods, each method having its own advantages and limitations.^1^ The first type, the quickest but less efficient in terms of organ transparency, includes techniques based on the simple immersion of the organ in a solution with high refractive index. The second and third types include techniques based on delipidation followed by either dehydration or hyperhydration, respectively, before RI matching. These techniques are efficient, relatively short, easily available especially in terms of material and costs. However, hyperhydration generates the swelling of the tissue and dehydration generates the shrinkage of the organ and, when combined with fluorescent reporter gene assay, fluorescence quenching. The fourth type includes techniques based on hydrogel embedding followed by delipidation before refractive index matching. These techniques are highly effective, highly conservative for organ structure and proteins, but long and difficult to set. Choosing the best clearing technique depends on the labeling procedure the experimenter wants to use to identify the object of interest. A recent review catalogs many procedures and associated optical clearing techniques to help choosing the best combination.^2^

Once the vascular network is stained and the organ optically cleared, 3D imaging can proceed. Basically, three 3D microscopy imaging techniques are available, confocal microscopy, multi-photon microscopy and light-sheet microscopy. The choice of the best technique mainly depends on the sample size, the resolution needed to visualize the structure of interest, and the equipment accessibility in terms of availability and cost. For vascular networks, the most used one is light-sheet microscopy.^3^^,^^4^^,^^5^ It is really interesting for studying vascular networks as it allows the acquisition of large immersed optically cleared samples (cm scale), quickly, reducing phototoxicity, and with high resolution.^1^ The latest light-sheet microscopes, such as UltraMicroscope Blaze (Miltenyi Biotec, Germany), Lightsheet 7 (Zeiss, Germany), InVi SPIM Lattice Pro (Brucker, USA) or Cleared Tissue LightSheet (3i, USA) can reach a resolution up to 1 × 1× 3 μm (x×y×z). As for confocal microscopy, light-sheet microscopy has anisotropic resolution, i.e., (z) is different from (x) and (y),^6^ which should be taken into account for image processing.

### X-Ray imaging

Non-photonic imaging techniques such X-ray microtomography or nanotomography, in radiography, allows the reconstruction of the 3D structure of an object using X-rays to create, non-invasively, cross-sections at different angles. They can be used after radiopaque perfusion of the vessels to image large vascular networks.^7^^,^^8^ While less efficient than light-sheet microscopy to visualize small vessels as capillaries due to a poor signal/noise ratio, they can be useful to study large ones as arteries.^9^

## Image processing

**Figure 2:**
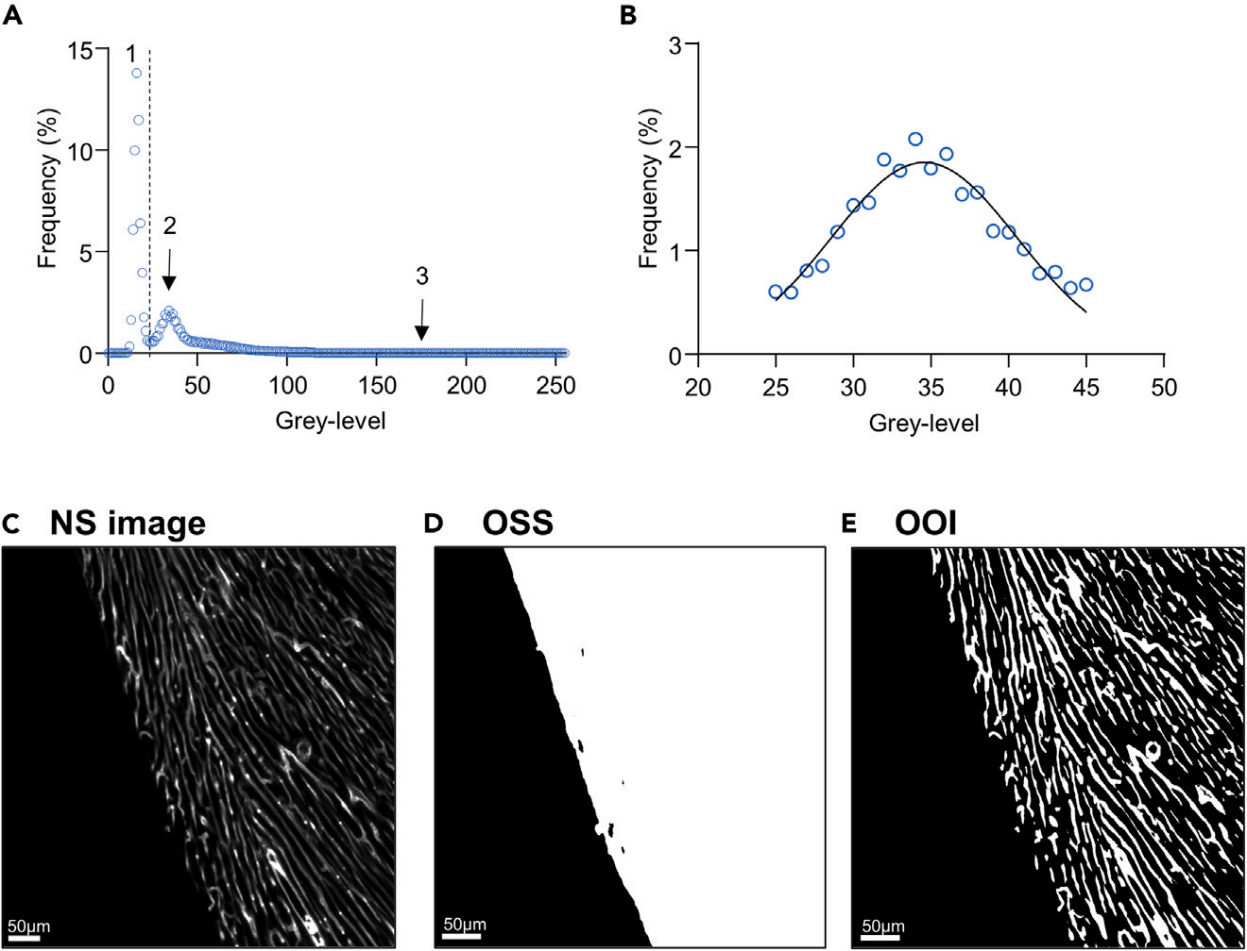
Gaussian fit thresholding technique principle (A) Representative frequency distribution of pixel population of lectin-labelled left ventricle after 3D imaging by light sheet microscopy. 1: background. 2: non-labelled cardiac tissue. 3: lectin-labelled capillary network. (B) Gaussian fit of the subset of pixels corresponding to the cardiac tissue from curve A. (C) Non segmented (NS) image of the left ventricle capillary network image obtained by light-sheet microscopy. (D) Object of study (OOS), full cardiac tissue, obtained by segmentation using the inflexion point technique between pixel population (1) and (2) from (C). (E) Object of interest (OOI), coronary capillary network, obtained by segmentation using the Gaussian fit technique on population (2) from (C).

Following the acquisition of the 3D image of the tissue (Figure 2C), image processing consists broadly of two steps, (1) the segmentation of the image, i.e., the extraction of the object of interest (OOI), that is, the vascular network from the background (Figure 2E), and (2) the skeletonization of the OOI, i.e., the identification of each segment and node of the network. Additionally, it is usually necessary to segment several relevant objects, such as (a) the vascular network (OOI) from the non-labeled tissue, and (b) the studied tissue (including the OOI) from the non-tissue background (e.g., the mounting medium) (Figure 2D). To avoid confusion between these 2 relevant objects, the studied tissue will be called “object of study” (OOS), the OOI being part of the OOS.

### Segmentation

#### General principles and limitations

Extraction of the OOI from the background actually combines two distinct objectives. One is the identification on the “correct” OOI. This is important to notice because, as seen later, several segmenting processes may select a “wrong” OOI. For this objective, eye-based evaluation by a skilled experimenter remains the reference. The other one is the delimitation of the OOI, i.e., the identification of its boundaries of edges. If, for qualitative imaging, the first objective is sufficient, for the quantitative characterization of the OOI, the accuracy of the determination of its boundaries is of critical importance.

The general principle of fluorescent (or radiopaque) labeling techniques of the vascular network is based on the fact (1) that the OOI is specifically labeled by the marker and (2) that the marker ensures a gray value higher than the background. As a result, the image supposedly contains two populations of voxels, i.e., three-dimensional pixels, one composed of high gray value voxels corresponding to the object of interest, contrasting with another one composed of low gray value voxels, itself usually composed of several subpopulations corresponding to the non-labelled tissue(s) and non-tissue objects (e.g., the mounting medium). The OOI can be hence segmented by setting a gray value threshold that separates the population of voxels corresponding to the OOI from that of the non-labelled background, then converting all the pixels with a gray-level equal or superior to the threshold into white pixels, and all the pixels with a gray-level inferior to it into black pixels. Thresholding hence creates a binary image, that is to say, for a 8-bit image, an image composed only of white (gray-level 255) and black pixels (gray-level 0), in which the vascular network appears in white on a black background.

Hence, grayscale-based thresholding ensures, in theory, the segmentation of the object of interest. The difficulty resides in the objective determination of this threshold, on which depends the objectivity of the subsequent quantitative analysis of the OOI. Indeed, these 2 populations are not separated form each other, but overlapping, as shown by the distribution curve of voxel grayscale values (Figure 2A).

The overlap can be explained by both practical and theoretical reasons. In practice, the sensitivity, the specificity and the homogeneity the labeling technique is not absolute, so that the labeling intensity of the structure of interest varies. Additionally, even if we hypothesize a “perfect” labeling, ensuring a homogeneous and maximal gray-level intensity of the object of interest, the voxelled image contains a various proportion of voxels with intermediate gray value. Indeed, since the gray value of a given voxel is the average value of the structures it contains, the voxels corresponding to the edge of the object of interest, hence including labeled and non-labelled structure, have intermediate gray value ranging from the highest gray value of the object of interest to the gray value of the background. An approximate calculation of the number of high level and intermediate voxels for a cylindric segment can be done as follows, considering a cylindric segment of length L and radius r, imaged with a voxel dimension d.

The total number of voxels corresponding to the segment volume ( $N_{v\left(V\right)}$) can be defined as the volume of the segment on the volume of the voxel, and is given by the following equation:

(Equation 1) $$N_{v\left(V\right)}=\frac{L\pi r^{2}}{d^{3}}$$

The number of voxels corresponding to the segment surface ( $N_{v\left(S\right)}$), i.e., the number of intermediate gray value voxels, can be defined as the surface of the segment on the surface of the voxel, and is given by the following equation:

(Equation 2) $$N_{v\left(S\right)}=\frac{L2\pi r}{d^{2}}$$

The proportion of intermediate gray value voxels on the total number of voxels is hence:

(Equation 3) $$\frac{N_{v\left(S\right)}}{N_{v\left(V\right)}}=\frac{L\pi r^{2}/d^{3}}{L2\pi r/d^{2}}=\frac{r}{2d}$$

The proportion of intermediate voxels depends hence on the voxel dimension and the size of each segment of the network, i.e., the vessel network structure, which is what the study wants to determine, so it is *a priori* unknown, making difficult to identify the voxel population corresponding to the OOI.

Objective setting of the threshold value is of critical importance for the quantitative analysis of the vascular network structure and comparison between experimental conditions, since its determines in practice the boundaries of the OOI and its subsequent quantitative characterization, which is very sensitive to the threshold value. We have calculated on two 3D images of the cardiac tissue, one of the capillary network obtained by LSM of lectin-labelled optically cleared hearts, and one of the coronary arterial network obtained by X-ray microtomography after radiopaque perfusion, that a variation of the threshold value by ±1 Gy value generated, a variation of ±18% and ±28% of the total segment number, ±21% and ±28% of the total junction number, and ±29% and ±14% of the volume density of the capillary and arterial vascular networks, respectively. Due to the overlap between the voxel populations of the OOI and the background, setting the threshold value is not trivial, and should be done with particular care. Over forty thresholding techniques have been described through the years, and categorized into six groups based on the information used by the algorithms to define the threshold.^10^ Thresholding techniques have been largely developed for human medical imaging techniques^11^^,^^12^ and are used mainly on 3D images obtained by X-ray microtomography or Magnetic Resonance Imaging. Scientific literature survey shows that the majority of the articles on 3D analysis of vascular networks in optically cleared organs use commercial software, allowing the automatic segmentation of the vascular network, regardless of the thresholding technique associated.^4^^,^^5^^,^^13^^,^^14^ In addition with commercial software, some laboratories have developed their own segmentation algorithms or computational tools to extract and quantify vasculatures.^6^^,^^15^^,^^16^^,^^17^ Finally, around fifteen thresholding techniques are available with the open-source freeware ImageJ, belonging to 3 categories of the Sezgin and Sankur’s classification, based on 1 the shape of the pixel gray-level histogram (the properties of the gray-level histogram such as its peaks and valleys), (2) the clustering analysis of the pixel gray-level histogram (the gray-level histogram is considered as corresponding to two clusters of pixels representing the object and the background), and (3) the entropy of the pixel gray-levels of the image (the image is considered as composed of “information” associated to the signal but mainly of noise, with a certain degree of uncertainty on which gray-level correspond to what). Also, some researchers have developed their own software to study a vascular network^8^ or methodologies,^18^ sometimes complex for a biologist to use. We have developed our own standardized thresholding method based on the statistical analysis of the voxel gray-level histogram, which can be performed using ImageJ plugins.^19^ We will present first this method, then summarize the principles of the most used thresholding plugin commercially available, Imaris software (Bitplane, Switzerland) FilamentTracer function, and finally the entropy-based automatic thresholding techniques, which are available with ImageJ.

#### Thresholding based on the Gaussian distribution of the background voxel gray-level histogram

The raw image usually contains non-tissue voxels (e.g., mounting medium) that should be removed to obtain the tissue. The frequency distribution diagram shows that the non-tissue voxel population is separated from the non-labelled tissue voxel population by an inflexion point that can be used as a threshold to segmentate the tissue (Figure 2A). Within the tissue, the segmentation of the labeled network is based on the analysis of the shape of the pixel gray-level histogram of the non-labeled background tissue (Figure 2B). As seen previously, the statistical characterization of the voxel population corresponding to the vascular network is not possible *a priori*, even considering a noise-free labeling. By contrast, the voxel population of non-labelled tissue, as far as it is homogeneous in term of transparency (or radiopacity), is easier to define and identify. Taking into account the interindividual variation inherent to any homogeneous population, it can be hypothesized that the gray-level frequency distribution of this non-labelled voxel population follows a Gaussian law. This can be verified by fitting with a Gaussian equation the frequency distribution of the background voxel subpopulation, which provides its mean and standard deviation (SD). Due to the general property of a Gaussian distribution, the threshold value can be defined as a confidence interval (CI) limit, defined as follow, $Z_{\alpha /2}$ being the critical score for a given α risk:

(Equation 4) $$CI=mean\pm Z_{\alpha /2}\times SD$$

For example, since $Z_{\alpha /2}$ = 1.96 for α = 0.05, setting the threshold at mean + 1.96SD ensures the exclusion of 97.5% of the voxels out of the OOI, so that the OOI contains always the same small proportion of background tissue in different samples, independently of any visual estimate of threshold setting. Since quantitative comparison between samples requires the OOI to be defined on the same quantitative basis, this thresholding technique is appropriate when the background tissue voxel frequency distribution actually follows a Gaussian law (additionally, if needed, OOS segmentation from non-tissue background can be done on the same principle by setting the threshold value at mean $-Z_{\alpha /2}$ SD). In practice, background voxels are usually heterogeneous, due to the fact that the raw images contain tissue (OOS) and non-tissue (e.g., mounting medium) voxels.

#### Thresholding using Imaris software FilamentTracer function

A survey of the literature on optical clearing and 3D imaging of vascular networks indicates that the most of the articles use, for the segmentation step, Imaris software (Bitplane, Switzerland) FilamentTracer function. It has have been used to isolate the vascular network in several organs such as the heart,^4^ the liver and the lung,^5^ the pancreas,^13^ the brachial and inguinal skeletal muscles,^14^ and the kidney.^9^ Imaris is a commercial software allowing 3D and 4D image analysis, and FilamentTracer is an extension initially allowing the automatic tracing of neurons and the detection of their spines, but extended for the tracing of any filamentous structures as blood vessels. Using FilamentTracer requires first to enter the diameter of the largest and thinnest vessels, usually estimated on 2D images. On this information, two threshold values are set automatically by the software to create the vessel skeleton. Then, a last threshold is automatically set to adjust the vessel diameter. These thresholds can be manually modified by the experimenter. FilamentTracer is easy to use, but its main weakness (in addition to the fact that the algorithm used to generate the threshold values is unknown by the experimenter) is that it is based on the initial visual, hence subjective, estimate of the vessel diameter by the experimenter and, sometimes, “ad-hoc” eye-based “correction” of the threshold values.

#### Entropy-based automatic thresholding techniques

These thresholding techniques are based on the entropy and the entropy power inequality, as defined by Claude Shannon in 1948 in his information theory, used to extract the useful signal (OOI) from white Gaussian noise.^20^ While using different methods for entropy power calculation, they are based on the same fundamental principle that the noise is spread uniformly, all over the image whereas the high energy signal is localized and represents a small part of the whole image. It has been shown that if the signal power is low compared to the noise it is absorbed by the Gaussian noise. The entropy power is hence the sum of the power of the noise and that of the signal, and, according to the principle of maximum entropy, the maximum entropy corresponds to the less informative distribution of the signal. This allows the determination of the Gaussian distribution of the noise and its deviation (σ), from which the threshold value can be set as σ.κ, κ being chosen from the probability of normal distribution. For example, it has been suggested to choose κ = 4 in order to exclude almost all of the noise.^20^

**Figure 3:**
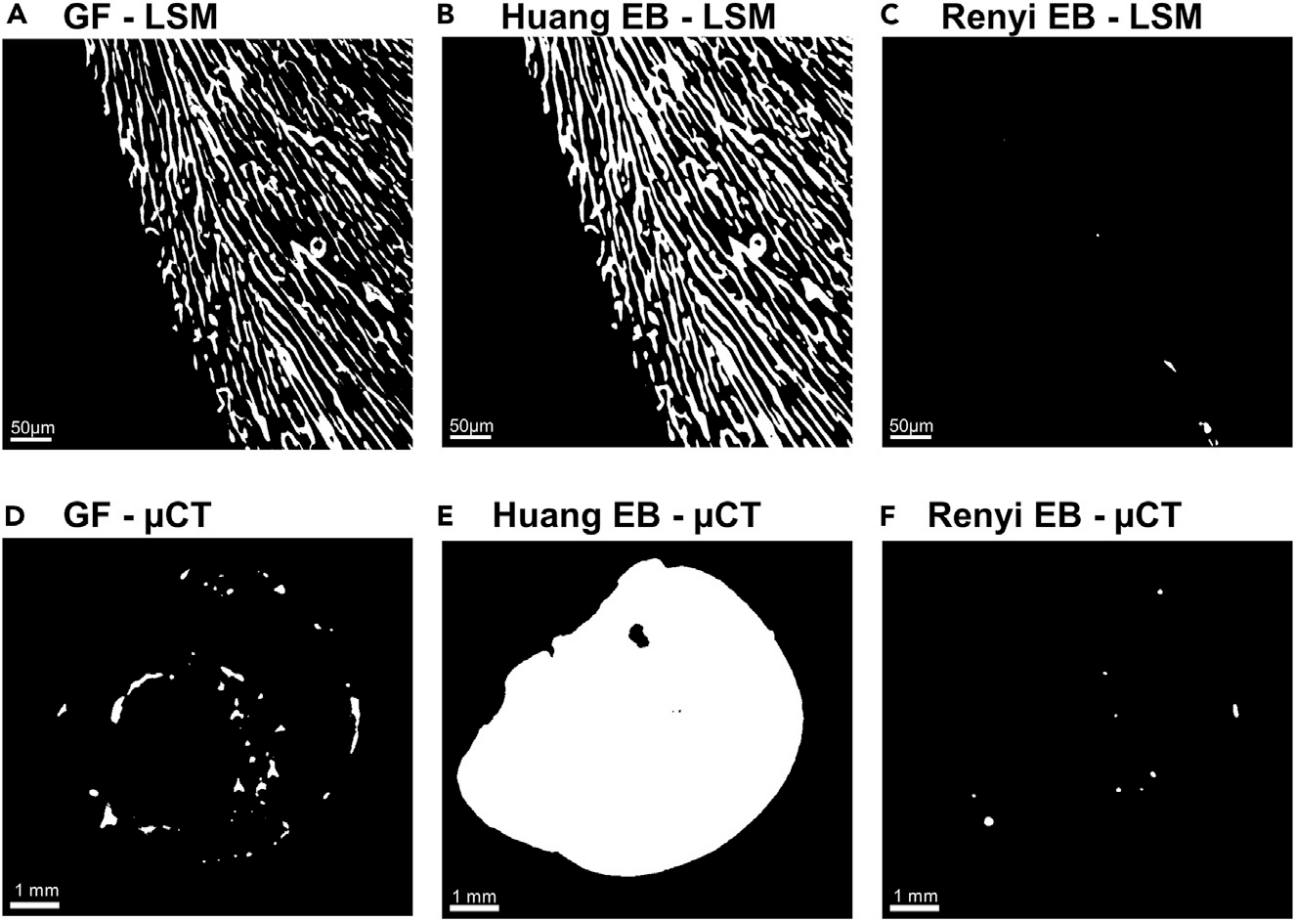
Comparitive segmentation between Gaussian fit (GF) and entropy-based (EB) techniques on light-sheet microscopy (LSM) and X-ray microtomography images (μCT) (A–F) (A, B, and C) LSM images of coronary capillary network. (D, E, and F) µCT images of coronary arterial network. (A, D) Images segmented by GF technique. (B, E) Images segmented by Huang EB technique. (C, F) Images segmented by Renyi EB technique.

These techniques are “neutral” regarding what is the OOI and “unsupervised,” i.e., the segmentation process is operator-independent. It can be seen as an advantage in terms of objectivity, but since several entropy-based applications are available and do not produce similar OOI segmentation, the operator should choose the “best” one, with the question of objective criteria defining what is “best.” Additionally, the neutrality may be problematic because the interest of the experimenter is not neutral regarding the nature of the studied object. Both OOI and OOS are of interest and should be extracted separately from the same image and the same noise+signal distribution, which a neutral technique cannot perform. Several thresholding functions are available in ImageJ, either entropy-based on not. We have tested all of them, using 2 images of the cardiac tissue, one of the capillary networks as OOI, obtained by light-sheet microscopy 3D images of lectin-labelled optically cleared hearts, and one of the coronary arterial network as OOI, obtained by X-ray microtomography imaging after radiopaque perfusion. The most efficient functions were the entropy-based ones, but they were not equally relevant in identifying the correct OOI. Those more relevant thresholding functions for light-sheet microscopy capillary images^21^^,^^22^ were not for images of coronary arteries, and reciprocally for those relevant for X-ray microtomography,^23^^,^^24^ as illustrated in Figure 3.

In conclusion, non-subjective threshold segmentation is critical for the objective quantification of the OOI, but is not easy to achieve. Conceptually, two criteria usually confused must be validated separately. First, (1) the nature of the relevant object (e.g., OOI versus OOS) is not “neutral” and should be based on the experimenter’s aims and expertise, whereas (2) the edges of this object should be delimited by experimenter-independent techniques. On these criteria, for opposite reason, neither the FilamentTracer function nor the neutral and unsupervised entropy-based functions are fully satisfactory. Our own standardized thresholding method based on voxel distribution analysis combines (1) a non-neutral identification of the voxel populations of interest (OOI versus OOS versus non-tissue background) with (2) objective (operator-independent thresholding) segmentation of OOS and OOI and fulfills the above-mentioned criteria.

### Skeletonization

**Figure 4:**
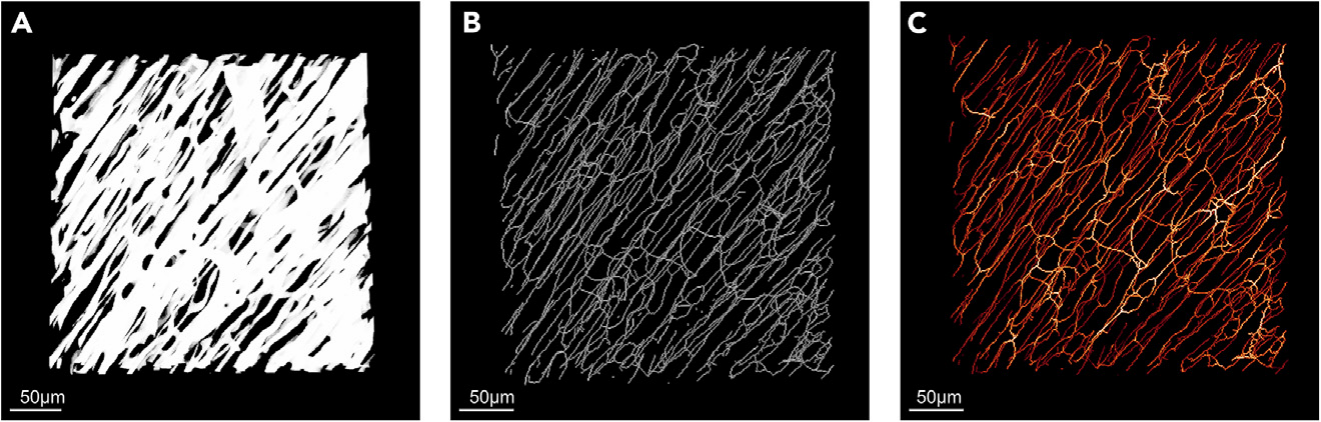
Image processing of left ventricle capillary network image obtained by light-sheet microscopy (A) Representative image segmented by Gaussian fit technique. (B) Skeleton of image (A). (C) Skeleton with distance mapping of image (A). Average radius of each segment are encoded in false colors from red (low) to white (high).

The skeletonization process consists in creating, from the segmented image of the network (Figure 4A), a thinner version of it, the “skeleton,” in which its segment is reduced to one pixel (2D) or voxel (3D) of diameter equidistant from the border of each segment (hence, centered) while conserving the topology (the same organization) and geometry (the same shape) of the object.^25^ A 3D skeleton is composed of a succession of one-voxel diameter segments delineated either by a junction or node, if the segment is connected to another one, or by an endpoint, if not. Multiple skeletonization algorithms exist that have been categorized in three approaches depending on the computational strategy used (Saha & al. in 2017). The ImageJ “Skeletonize (2D/3D)” function allowing the skeletonization of a 2D or 3D 8-bits binarized object is a digital approach consisting in the iterative symmetrical erosion of the object surface until a one-pixel/voxel centered skeleton remains (Figure 4B).^26^^,^^27^ This function allows the creation of a color-coded version of the skeleton for the easy visualization of the structure segments, junctions, and endpoints. More importantly, it allows the individual identification of each segment, defined by the orthonormal coordinates of its extremities, its length, and its Euclidean distance (linear distance between its extremities). Additionally, the mean radius of each segment can be extracted by coupling skeletonization and Euclidean distance mapping, a process consisting of the generation of the Euclidean distance map (EDM) of the structure showing, for each pixel/voxel), its shortest distance to a background pixel/voxel (Figure 4C).^28^ The ImageJ “3D Distance Map” function of the 3D Suite plugin^29^ can be used to generate EDM of 2D and 3D 8-bits binarized images, taking into account the calibration of the images, including anisotropic ones.

## Morphometric descriptors

This section presents the morphometric descriptors (MD) most frequently found in the literature and our own ones.^19^ These MDs are obtained from the skeleton analysis, which arises several issues. Due to the voxelization of the images and the resolution limit, in particular in the case of anisotropic resolution, skeleton analysis may generate erroneous centerline identification and length segment calculation, and artifactual node identification when performed on images without path smoothing.^30^^,^^31^^,^^32^^,^^33^ The following sections focus on MDs extractable following skeleton analysis using ImageJ “Analyze Skeleton (2D/3D)” tool, which taking into account anisotropy, but other tools specifically designed to address these issues have been recently developed, such as VesselVio^30^ and TubeMap.^32^

For practical reason, we have classified morphometric descriptors in 2 categories, (i) network-level MD, for which one value is obtained for each network, and (2) segment-level MD, for which one value is obtained for each segment. In the later case, due to the high number of segments per network, specific statistical analysis should be done to characterize the network as a whole. A summary of the literature about 3D MDs of several vascular networks after optical clearing is given in Table 1. As it can be seen, most of the studies are limited to network-level MD.

**Table 1:** Examples of morphometric descriptor (MD) values found in the literature

MD parameters	Value	Vessel type	Organ	Mouse strain	Vessel staining & Optical clearing (OC) methods	Imaging method	Software used	Reference
**Density**								

Volume^c^ (%)	34	Cap	LV	B6J	Lectin IV – iDisco+	LSM	ImageJ	Nicolas and Roux^19^
	19	Cap	S	B6J	Lectin IV – iDisco+	LSM	ImageJ	Nicolas and Roux^19^
	28	Cap	RV	B6J	Lectin IV – iDisco+	LSM	ImageJ	Nicolas and Roux^19^
	31	Cap	LV	B6J	Lectin IV – iDisco+	CM	ImageJ	Nicolas and Roux^19^
	12	HEVs	PLNs	B6J	MECA-79 AB – BABB	LSM	Imaris	Jeucken et al.^14^
Volume (μm^3^)	1.6 x 10^5^	Cap	IPIs	NOD	Lectin IV – No OC	CM	Imaris	El-Gohary et al.^13^
Surface (μm^2^)	4 x 10^−5^	BVs	Retina	B6J	CD31 AB – EyeCi	LSM	Imaris	Henning et al.^3^
Segments^d^	600,000	Cap	LV	B6J	Lectin IV – iDisco+	LSM	ImageJ	Nicolas and Roux^19^
	400,000	Cap	S	B6J	Lectin IV – iDisco+	LSM	ImageJ	Nicolas and Roux^19^
	390,000	Cap	RV	B6J	Lectin IV – iDisco+	LSM	ImageJ	Nicolas and Roux^19^
	4,300,000	Cap	LV	B6J	Lectin IV – iDisco+	CM	ImageJ	Nicolas and Roux^19^
Segments^e^	90	Cap	IPIs	NOD	Lectin IV – No OC	CM	Imaris	El-Gohary et al.^13^
Nodes^d^	290,000	Cap	LV	B6J	Lectin IV – iDisco+	LSM	ImageJ	Nicolas and Roux^19^
	170,000	Cap	S	B6J	Lectin IV – iDisco+	LSM	ImageJ	Nicolas and Roux^19^
	190,000	Cap	RV	B6J	Lectin IV – iDisco+	LSM	ImageJ	Nicolas and Roux^19^
	2,000,000	Cap	LV	B6J	Lectin IV – iDisco+	CM	ImageJ	Nicolas and Roux^19^
Nodes^e^	100	HEVs	PLNs	B6J	MECA-79 AB - BABB	LSM	Imaris	Jeucken et al.^14^
	55	Cap	IPIs	NOD	Lectin IV – No OC	CM	Imaris	El-Gohary et al.^13^
	190	BVs	Retina	B6J	CD31 AB - EyeCi	LSM	Imaris	Henning et al.^3^
Total length (μm)	13,000,000	Cap	LV	B6J	Lectin IV – iDisco+	LSM	ImageJ	Nicolas and Roux^19^
	11,500,000	Cap	S	B6J	Lectin IV – iDisco+	LSM	ImageJ	Nicolas and Roux^19^
	7,110,000	Cap	RV	B6J	Lectin IV – iDisco+	LSM	ImageJ	Nicolas and Roux^19^
	27,000,000	Cap	LV	B6J	Lectin IV – iDisco+	CM	ImageJ	Nicolas and Roux^19^
	1,000,000	HEVs	PLNs	B6J	MECA-79 AB - BABB	LSM	Imaris	Jeucken et al.^14^
	3,000	Cap	IPIs	NOD	Lectin IV – No OC	CM	Imaris	El-Gohary et al.^13^
	23,000	BVs	Retina	B6J	CD31 AB - EyeCi	LSM	Imaris	Henning et al.^3^
	1,600,000	BVs	Ovary	B6J	RG - CLARITY	LSM	Imaris	Oren et al.^5^

**Connectivity**								

Nodes/Segments	40,000	BVs	Ovary	B6J	RG – CLARITY	LSM	Imaris	Oren et al.^5^
	48%	Cap	LV	B6J	Lectin IV – iDisco+	LSM	ImageJ	Nicolas and Roux^19^
	43%	Cap	S	B6J	Lectin IV – iDisco+	LSM	ImageJ	Nicolas and Roux^19^
	49%	Cap	RV	B6J	Lectin IV – iDisco+	LSM	ImageJ	Nicolas and Roux^19^
	46%	Cap	LV	B6J	Lectin IV – iDisco+	CM	ImageJ	Nicolas and Roux^19^

**Architecture**								

Fractal Dimension	2.46	Cap	LV	B6J	Lectin IV – iDisco+	LSM	ImageJ	Nicolas and Roux^19^
	2.32	Cap	S	B6J	Lectin IV – iDisco+	LSM	ImageJ	Nicolas and Roux^19^
	2.33	Cap	RV	B6J	Lectin IV – iDisco+	LSM	ImageJ	Nicolas and Roux^19^
	2.53	Cap	LV	B6J	Lectin IV – iDisco+	CM	ImageJ	Nicolas and Roux^19^
Diameter^a^ (μm)	4.8	Cap	LV	B6J	Lectin IV – iDisco+	LSM	ImageJ	Nicolas and Roux^19^
	3.8	Cap	S	B6J	Lectin IV – iDisco+	LSM	ImageJ	Nicolas and Roux^19^
	5.1	Cap	RV	B6J	Lectin IV – iDisco+	LSM	ImageJ	Nicolas and Roux^19^
	3.1	Cap	LV	B6J	Lectin IV – iDisco+	CM	ImageJ	Nicolas and Roux^19^
	6.4	BVs	Retina	B6J	CD31 AB - EyeCi	LSM	Imaris	Henning et al.^3^
Length^b^ (μm)	14.3	BVs	Ovary	B6J	RG – CLARITY	LSM	Imaris	Oren et al.^5^
	12.3	Cap	LV	B6J	Lectin IV – iDisco+	LSM	ImageJ	Nicolas and Roux^19^
	12	Cap	S	B6J	Lectin IV – iDisco+	LSM	ImageJ	Nicolas and Roux^19^
	12.9	Cap	RV	B6J	Lectin IV – iDisco+	LSM	ImageJ	Nicolas and Roux^19^
Straightness	0.9	BVs	Ovary	B6J	RG – CLARITY	LSM	Imaris	Oren et al.^5^
Tortuosity^b^	1.2	Cap	LV	B6J	Lectin IV – iDisco+	LSM	ImageJ	Nicolas and Roux^19^
	1.2	Cap	S	B6J	Lectin IV – iDisco+	LSM	ImageJ	Nicolas and Roux^19^
	1.3	Cap	RV	B6J	Lectin IV – iDisco+	LSM	ImageJ	Nicolas and Roux^19^

OC, optical clearing; LV, cardiac left ventricle; RV, cardiac right ventricle; S, cardiac interventricular septum; PLNs, pair lymph nodes (brachial and inguinal); Cap, capillary; BVs, blood vessels; HEVs, high endothelial venules; B6J, C57BL6/J; IPIs, intrapancreatic islets; IV, intravenous injection; AB, antibody; RG, Vecad^cre^ x tdTomato^flox/stop/flox^ reporter gene; LSM, light-sheet microscopy; CM, confocal microscopy.

All MDs were obtained on wild type mice. Parameter values are given as rounded mean.

a Parameter value is the mean obtained by the frequency distribution after a non-linear regression of the date by a Gaussian curve.

b Parameter values are given as rounded median.

c Normalized per mm^3^ of the tissue studied.

d Number per mm^3^ the tissue studied.

e Number per studied zone.

### Network-level morphometric descriptors

#### Normalized volume, surface, node number, segment number and total length

These MD provide information regarding the density of the vascular network, but not on its architectural pattern. Le volume and surface of the vascular network can be obtained from its binary image without skeleton analysis. Skeleton analysis provides the total number of nodes and segments with their length, which sum correspond to the total length of the vascular network. This allows the calculation of the node number, segment number, and total length. For quantitative comparison, these parameters should be normalized to the volume of the perfused tissue (non labeled tissue + labeled vascular network), which requires, at explained previously, the segmentation of the tissue from the non-tissue background. Such morphometric descriptors can be used to characterize the microvascular rarefaction.

#### Node/segment ratio and furcation types

These MDs provide information regarding the connectivity of the vascular network, which can be characterized by all types of parameters that give information about the way the network segments are branched one on another, and the type of those branching points. Segments can be connected by different degrees of furcation defined by the number of segments connected to a given node (e.g., bifurcation = 3 segments, trifurcation = 4 segments). Theoretically, the relationship between the number of segments and of nodes depends on the degree of furcation of each node. For a non-hierarchical closed (without endpoints) meshwork as shown in Figure 1B, the number of segments is given by the following equation:

(Equation 5) $$S=\frac{1}{2}\left(\sum_{i=2}^{m}N_{\left(i\right)}\times i\right)$$

where S is the number of segments, *i* the degree of furcation (e.g., i = 3 means 3 segments connected to the same node), *m* the maximal degree of furcation, and *N(i)* the number of nodes of i-furcation. Hence, the node number/segment number ratio is an index of the average degree of furcation. Additionally, ImageJ skeleton analysis provides the number of bifurcations and trifurcations, which can be normalized to the total number of nodes, and expressed as %. Such MDs can be used as markers of alteration of vascular morphogenic processes. More advanced methods for network connectivity analysis, such as the network theory, have been developed and applied to vascular systems, but are beyond the scope of this article.^34^

#### Fractal dimension

As seen previously, vascular networks can be considered as fractal-like structures. The fractal dimension is a very interesting MD to characterize the architecture of the vascular network, as it expresses its ability fill space it occupies independently of its volume. Fractal analysis has been applied to a variety of as self-similar biological structures, including vascular and respiratory networks, for example to identify the effect of morphogenic genes^35^ or pathological alterations.^36^ Actually, if the fractal nature of the arterial network has already been shown, the fractal nature of the capillary network has not been yet formally proven.^37^ However, this does not prohibit to calculate its fractal dimension, and fractal analysis has been shown to be relevant in physiopathological studies, such as the development of tumor vascularization.^38^ The calculation of the fractal dimension from the vascular skeleton can be done automatically with ImageJ “BoneJ” plugin^39^ that uses the Minkowski-Bouligand “box counting” method.^40^

### Segment-level morphometric descriptors

#### Length, diameter and straightness or tortuosity

As said previously, skeletonization combined with distance mapping allows the identification of each segment of the studied network with the orthonormal coordinates of its extremities, its linear length (i.e., the Euclidean distance between its two extremities), its real length, and its radius., These parameters can be extracted using ImageJ, supplemented with the 3D Suites plugin for the radius.^29^ Hence, 3 main MDs can be easily obtained for each segment, its length, its diameter and its tortuosity or straightness. The diameter corresponds to 2 times the radius of the segment, expressed in length unit. The length correspond to the real length of each segment, expressed in length unit. The straightness corresponds to the ratio of the linear length of each segment on its real one, without unit, whereas the tortuosity is the inverse of the straightness. Maximal straightness and minimal tortuosity are equal to 1, for a straight segment.

#### Data analysis

**Figure 5:**
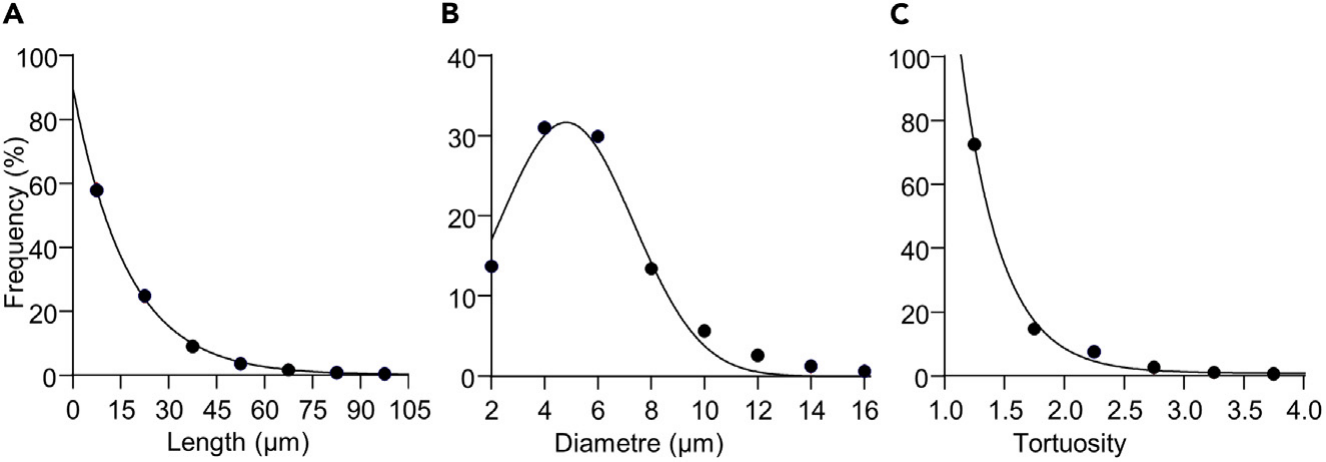
Non-linear regression analysis of the frequency distribution of network-level morphometric descriptors Data obtained from the left ventricle coronary capillary network of mapped skeleton analysis. (A) Segment length distribution fitted by first order exponential decay. (B) Segment diameter distribution fitted by Gaussian law. (C) Segment tortuosity distribution fitted by first order exponential decay.

For network-level MDs, one value is obtained for each network, so that each MD obtained on a sample of several networks can be statistically described by a summary value (e.g., mean, median) and individual dispersion (e.g., standard deviation), as usually done. For segment-level MDs, data on a single network are usually obtained from multiple thousands of segments and nodes. For each studied network, each segment-level MD can be summarized by a single value (e.g., mean or median), but it is poorly relevant, as it looses the informative richness provided by single-segment analysis. A more informative analysis is the frequency distribution of the MD value in each network and its fit by non-linear regression analysis. This provides a more accurate description of the population of segments than a single summarizing value. Additionally, it allows statistical comparison between samples and possible identification of how the network architecture may differ between samples. Several authors have proposed to use power law equation to fit frequency distribution of MDs of vascular and pulmonary networks because of their fractal structure.^36^^,^^41^^,^^42^ However, parameter distributions of self-similar structures do not necessarily follow a power law.^36^ We hence propose to fit each MD frequency distribution with the most accurate fitting law. We found that the frequency distribution of capillary segments length and tortuosity usually followed a first order exponential decay, whereas that of capillary radius followed a Gaussian law, as illustrated in Figure 5.

### Conclusion

Several 3D imaging techniques, image processing methods and MDs are available for quantitative 3D morphological study of vascular networks that can be applied to different organs. Some of them can be easily performed by biologists using ImageJ open-access freeware. However, rigorous quantitative analysis requires an explicit flow work that avoid subjective choice at any step. A key point of this flow work is the segmentation process, for which experimenter-dependent and independent steps should be clarified, distinguishing the identification of each OOI that depends on the experimenter’s choice and expertise, and the delimitation of the edges of each OII, which should be experimenter-independent. Analysis of different OOI on the same organ may request different imaging techniques, labeling methods and/or image processing. The informative richness of segment-level MDs is ensured by frequency distribution analysis rather than summarized by a single average value. Definition of standard MDs applicable to different organs and imaging techniques would be useful for comparison between studies.

## References

[1] Costa E.C., Silva D.N., Moreira A.F., Correia I.J. (2019). Optical clearing methods: An overview of the techniques used for the imaging of 3D spheroids. Biotechnol. Bioeng..

[2] Zhu J., Liu X., Deng Y., Li D., Yu T., Zhu D. (2021). Tissue optical clearing for 3D visualization of vascular networks: A review. Vascul. Pharmacol..

[3] Henning Y., Osadnik C., Malkemper E.P. (2019). EyeCi: Optical clearing and imaging of immunolabeled mouse eyes using light-sheet fluorescence microscopy. Exp. Eye Res..

[4] Merz S.F., Korste S., Bornemann L., Michel L., Stock P., Squire A., Soun C., Engel D.R., Detzer J., Lörchner H. (2019). Contemporaneous 3D characterization of acute and chronic myocardial I/R injury and response. Nat. Commun..

[5] Oren R., Fellus-Alyagor L., Addadi Y., Bochner F., Gutman H., Blumenreich S., Dafni H., Dekel N., Neeman M., Lazar S. (2018). Whole Organ Blood and Lymphatic Vessels Imaging (WOBLI). Sci. Rep..

[6] Drees D., Scherzinger A., Hägerling R., Kiefer F., Jiang X. (2021). Scalable robust graph and feature extraction for arbitrary vessel networks in large volumetric datasets. BMC Bioinf..

[7] Linder M., Duplaa C., Couffinhal T., Malandain G. (2015). 2015 IEEE 12th International Symposium on Biomedical Imaging (ISBI).

[8] Peeters G., Debbaut C., Laleman W., Monbaliu D., Vander Elst I., Detrez J.R., Vandecasteele T., De Schryver T., Van Hoorebeke L., Favere K. (2017). A multilevel framework to reconstruct anatomical 3D models of the hepatic vasculature in rat livers. J. Anat..

[9] Epah J., Pálfi K., Dienst F.L., Malacarne P.F., Bremer R., Salamon M., Kumar S., Jo H., Schürmann C., Brandes R.P. (2018). 3D Imaging and Quantitative Analysis of Vascular Networks: A Comparison of Ultramicroscopy and Micro-Computed Tomography. Theranostics.

[10] Sankur B., Sankur B. (2004). Survey over image thresholding techniques and quantitative performance evaluation. J. Electron. Imaging.

[11] Daniel P.V., Nagaraj B. (2013).

[12] Maolood I.Y., Al-Salhi Y.E.A., Lu S. (2018).

[13] El-Gohary Y., Sims-Lucas S., Lath N., Tulachan S., Guo P., Xiao X., Welsh C., Paredes J., Wiersch J., Prasadan K. (2012). Three-Dimensional Analysis of the Islet Vasculature. Anat. Rec..

[14] Jeucken K.C.M., Koning J.J., van Hamburg J.P., Mebius R.E., Tas S.W. (2021). A Straightforward Method for 3D Visualization of B Cell Clusters and High Endothelial Venules in Lymph Nodes Highlights Differential Roles of TNFRI and -II. Front. Immunol..

[15] Bukenya F., Nerissa C., Serres S., Pardon M.-C., Bai L. (2020). An automated method for segmentation and quantification of blood vessels in histology images. Microvasc. Res..

[16] Hägerling R., Drees D., Scherzinger A., Dierkes C., Martin-Almedina S., Butz S., Gordon K., Schäfers M., Hinrichs K., Ostergaard P. (2017). VIPAR, a quantitative approach to 3D histopathology applied to lymphatic malformations. JCI Insight.

[17] Todorov M.I., Paetzold J.C., Schoppe O., Tetteh G., Shit S., Efremov V., Todorov-Völgyi K., Düring M., Dichgans M., Piraud M. (2020). Machine learning analysis of whole mouse brain vasculature. Nat. Methods.

[18] Tsai P.S., Kaufhold J.P., Blinder P., Friedman B., Drew P.J., Karten H.J., Lyden P.D., Kleinfeld D. (2009). Correlations of Neuronal and Microvascular Densities in Murine Cortex Revealed by Direct Counting and Colocalization of Nuclei and Vessels. J. Neurosci..

[19] Nicolas N., Roux E. (2021). 3D Imaging and Quantitative Characterization of Mouse Capillary Coronary Network Architecture. Biology.

[20] Luthon F., Liévin M., Faux F. (2004). On the use of entropy power for threshold selection. Signal Process..

[21] Huang L.-K., Wang M.J.J. (1995). IMAGE THRESHOLDING BY MINIMIZING THE MEASURES OF FUZZINESS. Pattern Recoonition.

[22] Li C.H., Lee C.K. (1993). Minimum cross entropy thresholding. Pattern Recognit.

[23] Kapur J.N., Sahoo P.K., Wong A.K.C. (1985). A new method for gray-level picture thresholding using the entropy of the histogram. Comput. Vis. Graph Image Process.

[24] Yen J.C., Chang F.J., Chang S. (1995). A new criterion for automatic multilevel thresholding. IEEE Trans. Image Process..

[25] Saha P.K., Borgefors G., Sanniti di Baja G. (2017). Skeletonization.

[26] Homann H. (2007). Implementation of a 3D thinning algorithm. Insight J..

[27] Lee T.C., Kashyap R.L., Chu C.N. (1994). Building Skeleton Models via 3-D Medial Surface Axis Thinning Algorithms. CVGIP Graph. Models Image Process..

[28] Danielsson P.-E. (1980). Euclidean distance mapping. Comput. Graph. Image Process..

[29] Ollion J., Cochennec J., Loll F., Escudé C., Boudier T. (2013). TANGO: a generic tool for high-throughput 3D image analysis for studying nuclear organization. Bioinformatics.

[30] Bumgarner J.R., Nelson R.J. (2022). Open-source analysis and visualization of segmented vasculature datasets with VesselVio. Cell Rep. Methods.

[31] Chen Y., Zhang J., Wang S. (2009).

[32] Kirst C., Skriabine S., Vieites-Prado A., Topilko T., Bertin P., Gerschenfeld G., Verny F., Topilko P., Michalski N., Tessier-Lavigne M., Renier N. (2020). Mapping the Fine-Scale Organization and Plasticity of the Brain Vasculature. Cell.

[33] Palágyi K., Tschirren J., Hoffman E.A., Sonka M. (2006). Quantitative analysis of pulmonary airway tree structures. Comput. Biol. Med..

[34] Gosak M., Milojević M., Duh M., Skok K., Perc M. (2022). Networks behind the morphology and structural design of living systems. Phys. Life Rev..

[35] Markovič R., Peltan J., Gosak M., Horvat D., Žalik B., Seguy B., Chauvel R., Malandain G., Couffinhal T., Duplàa C. (2017). Planar cell polarity genes frizzled4 and frizzled6 exert patterning influence on arterial vessel morphogenesis. PLoS One.

[36] Tanabe N., Sato S., Suki B., Hirai T. (2020). Fractal Analysis of Lung Structure in Chronic Obstructive Pulmonary Disease. Front. Physiol..

[37] Chen Y. (2015). Fractals and Fractal Dimension of Systems of Blood Vessels: An Analogy between Artery Trees, River Networks, and Urban Hierarchies. Fractal Geom. Nonlinear Anal. Med. Biol..

[38] Craciunescu O., Das S.K., Dewhirst M.W. (1999). Advances in Heat and Mass Transfer in Biotechnology.

[39] Doube M., Kłosowski M.M., Arganda-Carreras I., Cordelières F.P., Dougherty R.P., Jackson J.S., Schmid B., Hutchinson J.R., Shefelbine S.J. (2010). BoneJ: Free and extensible bone image analysis in ImageJ. Bone.

[40] Luppe M. (2015). Fractal dimension based on Minkowski-Bouligand method using exponential dilations. Electron. Lett..

[41] Glenny R.W., Robertson H.T. (1990). Fractal properties of pulmonary blood flow: characterization of spatial heterogeneity. J. Appl. Physiol..

[42] Weibel E.R. (1991). Fractal geometry: a design principle for living organisms. Am. J. Physiol..

